# Brain areas involved with obsessive-compulsive disorder present different DNA methylation modulation

**DOI:** 10.1186/s12863-021-00993-0

**Published:** 2021-10-30

**Authors:** Kátia Cristina de Oliveira, Caroline Camilo, Vinícius Daguano Gastaldi, Arthur Sant’Anna Feltrin, Bianca Cristina Garcia Lisboa, Vanessa de Jesus Rodrigues de Paula, Ariane Cristine Moretto, Beny Lafer, Marcelo Queiroz Hoexter, Euripedes Constantino Miguel, Mariana Maschietto, Biobank for Aging Studies Group, Érika Dionisio Akiyama, Lea Tenenholz Grinberg, Renata Elaine Paraizo Leite, Claudia Kimie Suemoto, Renata Eloah de Lucena Ferretti-Rebustini, Carlos Augusto Pasqualucci, Wilson Jacob-Filho, Helena Brentani

**Affiliations:** 1 Departamento & Instituto de Psiquiatria, Faculdade de Medicina FMUSP, Universidade de Sao Paulo, Rua Dr. Ovídio Pires de Campos, 785 – LIM23 (Térreo), São Paulo, 05403-010 Brazil; 2 Center of Mathematics, Computation and Cognition, Federal University of ABC, São Bernardo do Campo, Brazil; 3 Faculdade de Medicina FMUSP, Universidade de Sao Paulo, Sao Paulo, Brazil; 4 Laboratório de Psicopatologia e Terapêutica Psiquiátrica (LIM23), Faculdade de Medicina FMUSP, Universidade de Sao Paulo, Sao Paulo, Brazil; 5 Centro de Pesquisa, Centro Infantil Boldrini, Campinas, Brazil; 6 Department of Neurology, University of California San Francisco, San Francisco, USA; 7 Escola de Enfermagem, Universidade de São Paulo, São Paulo, Brazil

**Keywords:** DNA methylation, Epigenetic age, Gene expression, Obsessive-compulsive disorder, Postmortem brain tissues

## Abstract

**Background:**

Obsessive-compulsive disorder (OCD) is characterized by intrusive thoughts and repetitive actions, that presents the involvement of the cortico-striatal areas. The contribution of environmental risk factors to OCD development suggests that epigenetic mechanisms may contribute to its pathophysiology. DNA methylation changes and gene expression were evaluated in post-mortem brain tissues of the cortical (anterior cingulate gyrus and orbitofrontal cortex) and ventral striatum (nucleus accumbens, caudate nucleus and putamen) areas from eight OCD patients and eight matched controls.

**Results:**

There were no differentially methylated CpG (cytosine-phosphate-guanine) sites (DMSs) in any brain area, nevertheless gene modules generated from CpG sites and protein-protein-interaction (PPI) showed enriched gene modules for all brain areas between OCD cases and controls. All brain areas but nucleus accumbens presented a predominantly hypomethylation pattern for the differentially methylated regions (DMRs). Although there were common transcriptional factors that targeted these DMRs, their targeted differentially expressed genes were different among all brain areas. The protein-protein interaction network based on methylation and gene expression data reported that all brain areas were enriched for G-protein signaling pathway, immune response, apoptosis and synapse biological processes but each brain area also presented enrichment of specific signaling pathways. Finally, OCD patients and controls did not present significant DNA methylation age differences.

**Conclusions:**

DNA methylation changes in brain areas involved with OCD, especially those involved with genes related to synaptic plasticity and the immune system could mediate the action of genetic and environmental factors associated with OCD.

**Supplementary Information:**

The online version contains supplementary material available at 10.1186/s12863-021-00993-0.

## Background

Obsessive-compulsive disorder (OCD) is a debilitating neurodevelopmental condition that affects up to 3% of the worldwide population according to the World Health Organization [1]. OCD is characterized by intrusive thoughts and repetitive behaviors in a large time-consuming manner [2, 3].

Cortical areas [anterior cingulate gyrus (ACC), dorsolateral prefrontal cortex (dlPFC) and orbitofrontal cortex (OFC)] maintain the main projections to the ventral striatum areas [nucleus accumbens (NAC), caudate nucleus (CN) and putamen (PT)] [2, 4], both involved with OCD symptoms, paradigms [5], and treatment response [6]. The cortico-striato-thalamo-cortical circuitry (CSTC) is altered in the brain of OCD individuals, which includes three relevant loopings of indirect pathways with the respective cortical connection: the affective (ACC, NAC and thalamus), dorsal cognitive (dlPFC, CN and thalamus) and ventral cognitive (OFC, PT and thalamus) circuits, which are related to the affective and reward processing, the working memory and executive function, and the motor and inhibitory response, respectively [2].

Neuroimaging MRI studies using whole-brain voxel-based morphometry (VMB) revealed that changes in anatomical structures from both affective and cognitive (executive) circuits are consistently described in OCD cases and were related to variation in symptom severity [7]. Diffusion-weighted magnetic resonance imaging was associated with gene expression alterations confirming the tripartite model of striatum organization and connection model [8]. We explored this model by using the differentially expressed (DEGs) and coexpressed genes modules in CN, NAC and PT brain tissues from OCD cases and controls, revealing the involvement of cell communication, cell response, synaptic transmission and plasticity for all striatum areas [9].

Different studies demonstrated that OCD etiology is a multifactorial condition with both polygenic and environmental risk factors [3, 10]. The impact of environmental factors may reflect changes in DNA methylation (DNAm), an epigenetic modification that consists in the addition of a methyl group (CH3) to carbon at the fifth position of cytosine (C) [11]. DNA methylation intermediates the interaction between genetic and environmental factors involved with psychiatric disorders [12]. Specifically for OCD, DNAm has been investigated in peripheral tissues, such as blood [13–15] including mononuclear cells [16] and saliva [17]. Differentially methylated CpG (cytosine-phosphate-guanine) sites between OCD patients and controls were only partially able to group patients (67%) in an unsupervised clustering analysis. These CpG sites were located in genes enriched for actin cytoskeleton, cell adhesion molecules (CAMs), actin binding, transcription regulator activity, and other cellular pathways [13]. By evaluating methylation levels from selected CpG sites, no changes were observed in 14 genes previously associated with OCD [14]. However, *OXTR*, the oxytocin receptor gene, presented higher methylation in the OCD patients and correlated with severity and oxytocin was associated with the regulation of complex socio-cognitive processes [15]. DNAm levels of a CpG site located in the first intron from *SLC6A4* were higher in the saliva of pediatric and adult OCD patients compared to controls but no alteration was observed for *SLC6A4* expression [17]. The opposite way, higher methylation of two CpG sites located at *BDNF* exon 1 correlated with higher expression in OCD patients [16].

These data, derived from surrogate tissues, point to the necessity of exploring DNAm in the brain areas associated with OCD to verify its possible contribution for the disease. Furthermore, the methylation clock [18] in brain tissues from patients with OCD should be disclosed as it is a complement of tissue senescence. The DNA methylation clock is associated with physiological ageing but also is associated with changes according to stress exposition along life [19] and age acceleration was associated with other psychiatric disorders [20, 21].

Considering our small sample size to explore DNA methylation comparing cases and controls, to avoid false positive results, we performed gene network analysis of DNAm integrated with transcriptomic data in the brain areas associated with OCD.

## Results

### Characterization of OCD cases and controls

Individuals from both groups (OCD cases and controls) presented similar socio-demographic characteristics and were matched by sex and age. All individuals were older than 50 years and did not have a history of clinical dementia at the time of death **(**Table 1**)**.

**Table 1:** Demographic characteristics of obsessive-compulsive disorder (OCD) cases and controls

Variable	Parameters	*OCD* *N = 8*	*Controls* *N = 8*	*p-value*
Age (years)	Mean ± SD	76.4 ± 12.3	74.1 ± 13.6	0.73^†^
Sex, n (%)	Female	3 (37.5)	3 (37.5)	1.00^††^
	Male	5 (62.5)	5 (62.5)	
Education (years)	Mean ± SD	2.2 ± 2.8	5.5 ± 5.4	0.19^†††^
Alcoholism, n (%)	Yes	1 (12.5)	1 (12.5)	1.00^††^
	Never/Stopped	7 (87.5)	7 (87.5)	
Smoking, n (%)	Yes	3 (37.5)	3 (37.5)	1.00^††^
	Never/Stopped	5 (62.5)	5 (62.5)	
Brain volume (ml)	Mean ± SD	1098.3 ± 77.9	1283.4 ± 302.7	0.19^†††^
Brain weight (g)	Mean ± SD	1151.7 ± 106.3	1182.1 ± 123.2	0.65^†^
pH	Mean ± SD	7.1 ± 0.6	6.7 ± 0.3	0.24^†††^
Hemisphere, n (%)	Right	3 (37.5)	4 (50)	1.00^††^
	Left	5 (62.5)	4 (50)	
Post-mortem interval (min)	Mean ± SD	859.0 ± 179.3	891.1 ± 172.0	0.74^†^

^*†*^*t-test;* ^*††*^*Fisher’s Exact test;* ^*†††*^*Mann–Whitney U test (Confidence Interval – 95)*

### DNA methylation data comparing OCD cases and controls

For all brain areas, comparison between OCD cases and controls did not point to differentially methylated CpG sites (DMSs) after multiple correction tests (adjP≤0.05). Additional file 2**: Table S1** presents CpG sites with a *p*-value < 0.0005 for each brain area. The EpiMod algorithm, which is based only in methylation data and protein-protein-interaction (PPI) networks, from Functional Epigenetic Modules (FEM) analysis, infers differential methylation hotspots called modules. It showed enriched gene modules for all brain areas between OCD cases and controls: five modules for ACC, nine for OFC, five for NAC, eight for CN and three for PT **(**Additional file 2**: Table S2)**. ACC and PT modules were predominantly hypomethylated, NAC and CN were mostly hypermethylated and OFC had both hypermethylated and hypomethylated modules **(**Additional file 1**: Fig. S1)**.

Considering differentially methylated regions (DMRs), four out of five brain areas presented mostly a hypomethylation pattern: 70 DMRs for ACC (22 hypermethylated and 48 hypomethylated), 356 for OFC (140 hypermethylated and 216 hypomethylated), 75 for CN (26 hypermethylated and 49 hypomethylated), 106 for PT (11 hypermethylated and 95 hypomethylated). Only NAC had more hypermethylated than hypomethylated DMRs (*n* = 174, 138 hypermethylated and 36 hypomethylated) **(**Additional file 2**: Table S3)**. Some genes located in DMRs were also identified by EpiMod-FEM analyses, such as *HLA-DQB1* and *HLA-DQA1.* DMRs located at *ADARB2* and *UGT2B15* were hypermethylated in OFC DMSs, but do not survive after the multiple test correction (*p*-value: 1.33E-05/ Δβ: 0.186; p-value: 3.60E-04/ Δβ: 0.208, respectively).

### DNA methylation and gene expression integration data

**Fig. 1:**
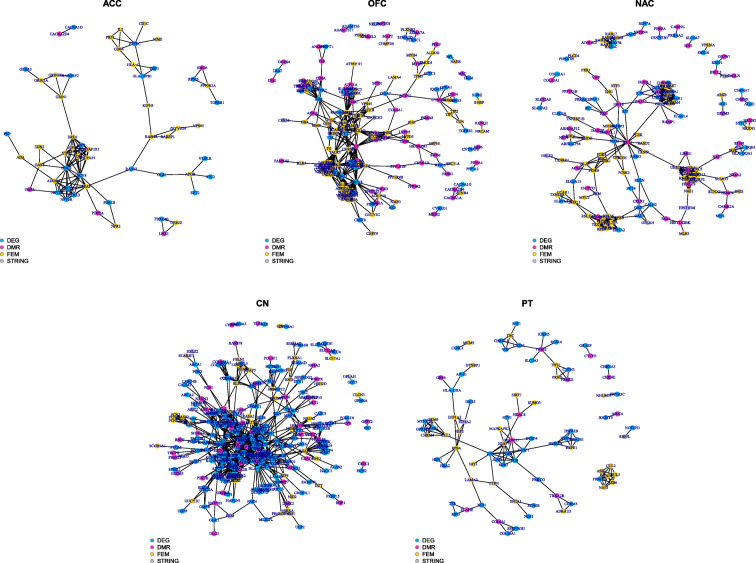
PPI networks from STRING [86] for the five brain areas using DEG, genes with DMRs and genes from FEM modules. Network plots were created using the igraph (v.1.2.4.2) library

As we have a small sample size, methylation and transcriptomic data integration from the same brain areas from OCD cases and controls resulted in a more robust approach. The list of differentially expressed genes (DEGs) comparing OCD cases and controls from NAC, CN and PT was retrieved from a previously published study by our group [9]. To search DEGs for ACC and OFC, RNASeq data were preprocessed using the same parameters as described in the methods section. First, data were integrated by using genes from DMRs associated with genes, FEM enriched modules from methylation and DEGs from the RNASeq data (*p* < 0.01) **(**Additional file 2**: Table S4)** to construct a PPI network for each brain area. To achieve a more accurate analysis regarding connected transcriptomic and methylation data, we selected only edges that connected nodes from different lists, e.g. one node from the DEG list and another from the DMR or FEM lists. All nodes were classified by network centrality measures **(**Additional file 2**: Table S5)** and by using a 95 percentile as threshold for each topological measure ranked list, we observed that genes from DEGs, DMRs and FEM are represented. For OFC PPI, 40 (23%), 51 (29%), 86 (48%); from NAC PPI, 44 (29%), 41 (27%), 69 (45%); from CN PPI, 186 (66%), 51 (18%) and 46 (16%); and for PT PPI, 44 (53%), 11 (13%) and 28 (34%) **(**Fig. 1**)**. Interestingly, *HLA-DQA1*, reported as differentially methylated in CN DMR and FEM analyses, had a high classification in the rank according to its degree and closeness measures in the network. Searching for genes previously associated with OCD in the networks, all brain areas have specific genes as well as genes represented in more than one brain area **(**Table 2**)**.

**Table 2:** Genes (nodes) belonging to the final networks that were already associated with OCD

Brain area	Code	Gene	Function ^†^
**ACC**	CNVs [91]	*NPY1R*	Belongs to the G-protein-coupled receptor superfamily; Nervous system and immune system phenotype; Behavior/neurological phenotype; Mortality/aging.
		*NPY5R*	Belongs to the G-protein-coupled receptor superfamily; Behavior/neurological phenotype.
		*GRIN1*	Related to neurodevelopmental disorder; Relation with schizophrenia; Polymicrogyria.
		*RASGRF2*	T-cell signaling response; Related to Alchoolism.
	GWAs [45]	***HLA-DPB1*** ^**††**^	**Binds peptides derived from antigens that access the endocytic route of antigen presenting cells and presents them on the cell surface for recognition.**
		***ADCY8*** ^**††**^	**Catalyses the formation of cyclic AMP from ATP; Increase cyclic adenosine monophosphate (cAMP) levels, resulting in the transcriptional activation of target genes; Related to mood disorder.**
	CNVs/	*TGFBR1*	Transduces the TGFB1, TGFB2 and TGFB3 signal from the cell surface to the cytoplasm and is thus regulating a plethora of physiological and pathological processes including cell cycle arrest.
	Exome [92]	*UBE2Z*	Encodes an enzyme which ubiquitinates proteins which participate in signaling pathways and apoptosis; Innate Immune System.
		*RABEP1*	Vesicle-mediated transport.
**OFC**	CNVs	*CDH10*	Among its related pathways are ERK Signaling and Cell junction organization; GO annotations related to this gene include calcium ion binding; Mediate calcium-dependent cell-cell adhesion.
		*ASAH1*	ASAH1 silencing increased basal and cAMP-dependent cortisol, establishing ASAH1 as a pivotal regulator of steroidogenic capacity in the human adrenal cortex.
		*ENTPD2*	Among its related pathways are ATP/ITP metabolism and metabolism of nucleotides.
		*YES1*	Encoded protein has tyrosine kinase activity and belongs to the src family of proteins.
		*IL17RD*	Encodes a membrane protein belonging to the interleukin-17 receptor (IL-17R) protein family, a component of the interleukin-17 receptor signaling complex.
		*TACR3*	Belongs to a family of genes that function as receptors for tachykinins, characterized by interactions with G proteins.
		*VTI1B*	SNARE protein.
	GWAs	*CHMP2B*	Expressed in neurons of all major regions of the brain; Mutations in this gene result in one form of familial frontotemporal lobar degeneration.
		*PDE4D*	Hydrolyzes the second messenger cAMP, which is a key regulator of many important physiological processes.
		*PPP1R9B*	Modulates excitatory synaptic transmission and dendritic spine morphology; Binds to actin filaments and shows cross-linking activity; Play an important role in linking the actin cytoskeleton to the plasma membrane at the synaptic junction; Plays a role in regulation of G-protein coupled receptor signaling; Related to schizophrenia.
		*SCARB2*	Acts as a lysosomal receptor for glucosylceramidase (GBA) targeting.
	Exome	*COL4A1*	Mutations in this gene cause porencephaly, cerebrovascular disease, and renal and muscular defects.
	mRNA [93]	*CACNB4*	Encodes a member of the beta subunit family of voltage-dependent calcium channel complex proteins. Related to epilepsy.
**NAC**	CNVs	***NAPB*** ^**††**^	**Associated with obsessive-compulsive personality disorder, amyotrophy, hereditary neuralgic and neurodegeneration with brain iron accumulation.**
		*PDK4*	Plays a role in cell proliferation via its role in regulating carbohydrate and fatty acid metabolism.
		***SLC5A7*** ^**††**^	**Transmembrane transporter that imports choline from the extracellular space into the neuron with high affinity.**
		*SLC2A13*	Transport related stereoisomers.
		*EPRS*	Multifunctional protein that catalyzes the attachment of the cognate amino acid to the corresponding tRNA; Microcephaly, progressive, with seizures and cerebral and cerebellar atrophy.
		*UBE2D1*	Mediates the selective degradation of short-lived and abnormal proteins.
		*SCG5*	Plays a role in regulating pituitary hormone secretion.
	GWAs	*KIT*	Encodes a receptor tyrosine kinase; Related with multiple intracellular proteins that play a role in in the proliferation, differentiation, migration and apoptosis of many cell types.
	Exome	*RAB25*	Member of the RAS superfamily of small GTPases; Involved in membrane trafficking and cell survival; Cytoskeletal Signaling; Metabolism of proteins.
		*RHOD*	Involved in endosome dynamics and reorganization of the actin cytoskeleton; Rho proteins interact with protein kinases and may serve as targets for activated GTPase.
		*RPL28*	Encodes one of the small GTP-binding proteins in the Rho family shown to be associated with focal adhesions in endothelial cells.
		*RHOJ*	Encodes one of the small GTP-binding proteins in the Rho family shown to be associated with focal adhesions in endothelial cells.
		*HERC5*	Pro-inflammatory cytokines upregulate expression of this gene in endothelial cells; Functions as an interferon-induced E3 protein ligase that mediates ISGylation of protein targets.
		*PRKAA2*	Catalytic subunit of the AMP-activated protein kinase (AMPK), a heterotrimer consisting of an alpha catalytic subunit, and non-catalytic beta and gamma subunits.
		*RAB13*	Member of the Rab family of small G proteins; Plays a role in neuronal regeneration and regulation of neurite outgrowth.
	mRNA	*RPL35*	Catalyze ribosomes, which consist of a small 40S subunit and a large 60S subunit and together are composed of 4 RNA species; rRNA processing in the nucleus and cytosol.
		*RPL6*	Encodes a protein component of the 60S ribosomal subunit; rRNA processing in the nucleus and cytosol.
**CN**	CNVs	*CSPG4*	May also inhibit neurite outgrowth and growth cone collapse during axon regeneration.
		*GPSM2*	Belongs to a family that modulate activation of G proteins; Required for cortical dynein-dynactin complex recruitment during metaphase.
		***PON3*** ^**††**^	**Childhood aggressive behaviour measurement; Immune system phenotype.**
		***LTBP1*** ^**††**^	**Key regulator of TGFB1, TGFB2 and TGFB3 that controls TGF-beta activation by maintaining it in a latent state during storage in extracellular space.**
		*WWOX*	Putative oxidoreductase; Acts as a tumor suppressor and plays a role in apoptosis; Multiple sclerosis.
		***ABCA2*** ^**††**^	**May have a role in macrophage lipid metabolism and neural development.**
		***CYFIP1*** ^**††**^	**Regulates formation of membrane ruffles and lamellipodia; Plays a role in axon outgrowth.**
		***CADM2*** ^**††**^	**Important for synapse organization, providing regulated trans-synaptic adhesion; Preferentially binds to oligodendrocytes.**
		*ELN*	Encodes a protein of elastic fibers, which comprise part of the extracellular matrix and confer elasticity to organs and tissues.
		*MLXIPL*	Encodes a basic helix-loop-helix leucine zipper transcription factor of the Myc/Max/Mad superfamily.
	GWAs	*SH3RF1*	Has E3 ubiquitin-protein ligase activity; Innate Immune System.
		***HLA-DPA1*** ^**††**^	**It plays a central role in the immune system by presenting peptides derived from extracellular proteins.**
	Exome	*C4B*	Encodes the basic form of complement factor 4, and together with the C4A gene, is part of the classical activation pathway; Innate Immune System.
		*NR0B2*	An unusual orphan receptor that contains a putative ligand-binding domain but lacks a conventional DNA-binding domain.
		*CALM1*	Encodes calmodulin proteins, members of calcium-binding protein family. Calcium-induced activation of calmodulin regulates and modulates the function of cardiac ion channels.
		*NFE2*	GO annotations related to this gene include DNA-binding transcription factor activity and transcription coactivator activity.
		*RNASE2*	Is a non-secretory ribonuclease that belongs to the pancreatic ribonuclease family, a subset of the ribonuclease A superfamily; Innate Immune System.
		*SERPINA1*	Inhibitor of serine proteases; Innate Immune System; Related to mental retardation, x-linked, associated with fragile site fraxe.
		*FBLN1*	Is a secreted glycoprotein that becomes incorporated into a fibrillar extracellular matrix; Cell adhesion; Degradation of the extracellular matrix.
		*DLG4*	Is recruited into NMDA receptor and potassium channel clusters; Intellectual developmental disorder; Presynaptic function of Kainate receptors.
		*DLG2*	Encodes a member of the membrane-associated guanylate kinase family; Protein-protein interactions at synapses; Tight junction; Related to autism disorder.
		*LYN*	Encodes a tyrosine protein kinase; B cell receptor signaling pathway (KEGG); Immune response Fc epsilon RI pathway.
**PT**	CNVs	*PRND*	Mutations in this gene may lead to neurological disorders; Association with sporadic Creutzfeldt-Jakob disease; Immune system phenotype.
		*MUC4*	May play a role in tumor progression.
	GWAs	*DTNBP1*	Plays a role in synaptic vesicle trafficking and in neurotransmitter release; May play a role in actin cytoskeleton reorganization and neurite outgrowth; May modulate MAPK8 phosphorylation; Appears to promote neuronal transmission and viability, modulating PI3K signaling and influencing glutamatergic release; Modulates prefrontal cortical activity via the dopamine/D2 pathway.
	Exome	*JUND*	Has been proposed to protect cells from p53-dependent senescence and apoptosis; MAPK signaling pathway.
		*AP1S1*	Protein encoded by this gene is part of the clathrin coat assembly complex which links clathrin to receptors in coated vesicles, involved in endocytosis and Golgi processing.
		*JUN*	Cognitive function measurement.
**ACC/ OFC**	CNVs	***ADCYAP1*** ^**††**^	**Related pathways are Signaling by GPCR and presynaptic function of Kainate receptors.**
		*CACNA2D4*	Encodes a protein in the voltage-dependent calcium channel complex; Related to bipolar disorder.
**OFC/ CN**	CNVs	*PON1*	Protein Coding gene; Diseases associated include microvascular complications of diabetes and amyotrophic lateral scclerosis.
	Exome	*C3*	Plays a central role in the activation of complement system. Adaptive Immune System
**OFC/ PT**	Exome / mRNA	***GBP4*** ^**††**^	**Are induced by interferon and hydrolyze GTP to both GDP and GMP; Cytokine Signaling in Immune system.**
**NAC/ CN**	Exome	*RASD2*	Belongs to the Ras superfamily of small GTPases and is enriched in the striatum. Encoded protein binds to mutant huntingtin (mHtt), mutated in Huntington disease (HD). Sumoylation of mHTT by this protein may cause degeneration of the striatum.
		*AKT1*	Protein kinase family; AKT/PI3K forms a key component of many signalling pathways; Regulate many processes including metabolism, proliferation, cell survival, growth and angiogenesis.
		*FAIM2*	Protein Coding gene; Regulates Fas-mediated apoptosis in neurons by interfering with caspase-8 activation; Disease associated includes Ventilation Pneumonitis and OCD.
**NAC/ CN/ PT**	CNVs	*CHRM5*	Belong to a larger family of G protein-coupled receptors and influence many effects of acetylcholine in the central and peripheral nervous system; Important for prolonged dopamine release; Related to schizophrenia.

^†^*Resumed from entrez gene cards (**https://www.genecards.org/**) and NCBI gene database (**https://www.ncbi.nlm.nih.gov/gene/**);* ^††^ *Genes in the 95 percentile are indicated in bold*

For each network, we searched for functional module enrichment analysis as described in the methods section, considering non-redundant ontologies/pathways with a minimum of 5 genes. PPI networks from all brain areas were enriched for G-protein signaling pathway, immune response, apoptosis and synapse biological processes. Also, all areas but CN were enriched for different behaviors, including feeding, learning and memory. ACC, OFC, NAC and CN were also enriched for axon, dendrite, purine process, response to stress, GTPase and MAPK activity. Regarding exclusive processes, ACC PPI network was enriched for cAMP signaling, OFC for inflammatory response and interferon-gamma signaling pathway, NAC for acetylcholine receptor activity and cholinergic synaptic transmission, and PT for transcriptional regulation. CN PPI network had the higher number of enriched processes, such as myelination, glial cell development, peripheral nervous system development, regulation of I-kappaB kinase/NF-kappaB signaling, PI3K signaling, Ras and Rho proteins signal transduction, type I interferon and JAK-STAT signaling pathways, JUN kinase activity, MHC class II receptor activity and regulation of transcription in response to stress.

Considering the REACTOME, GPCR signaling was enriched in all areas. NAC, CN and PT were also enriched for MAP kinase activity. Some areas have more specific pathways such as neurotransmitter receptors and postsynaptic signal transmission in ACC, interferon gamma signaling and MHC class II antigen in OFC, Rho GTPase cycle, interferon signaling, neddylation and pyruvate metabolism in NAC, interleukin-17 signaling, class I MHC processing, neddylation and cellular senescence in PT. CN was enriched for interferons gamma, alpha/beta signaling, interleukins 3, 4, 5, 10 and 13 signaling, Rho GTPases signaling, MHC class I and II antigen presentation, transcriptional regulation by RUNX family genes, NMDA and GABAB receptors **(**Additional file 2**: Table S6)**.

### DNA methylation and gene expression integration data from DMRs not located at genes

**Fig. 2:**
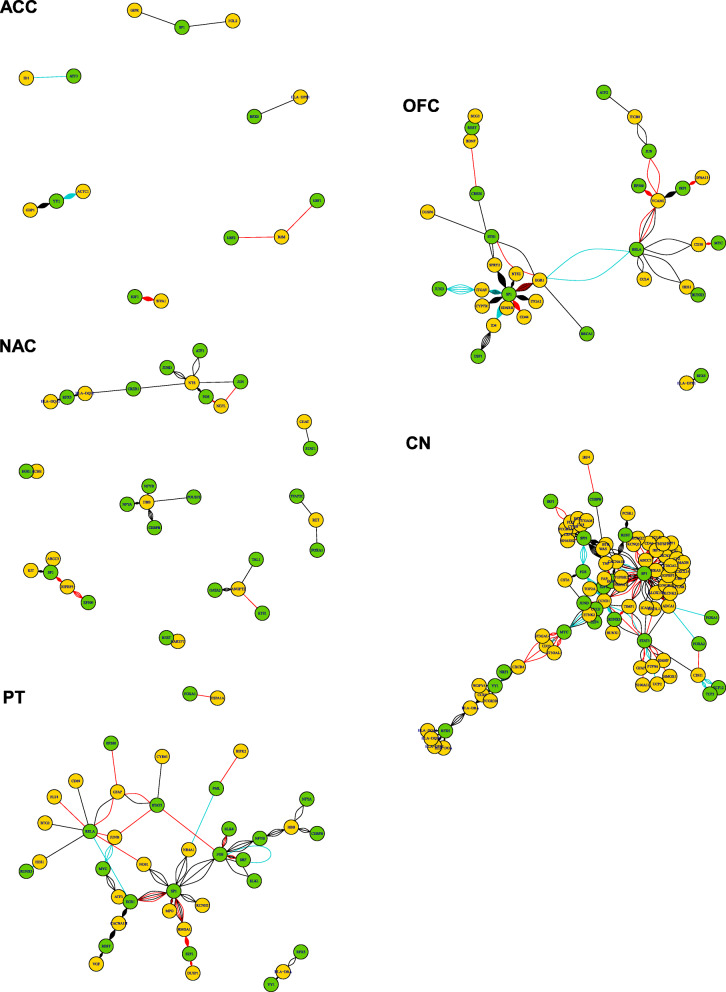
Regulatory Networks for TFs from DMRs and targeted DEGs for the five brain areas. Network plots were created using the igraph (v.1.2.4.2) library

To present a comprehensive assessment of DMRs, including those not located at genes, they were submitted to ENCODE [22] to be annotated to TFs. We identified seven TFs for ACC, 15 for OFC, 20 for NAC, 21 and 20 for CN and PT, respectively. Genes targeted by the TFs were searched within DEGs (Additional file 2: Table S7). In relation to TFs targeted DEGs, we identified eight for ACC, 19 for OFC, 13 for NAC, 68 for CN and 22 PT. TFs and their targeted DEGs as well as its activation/repression relation were used as connections to construct a network **(**Fig. 2**)**.

Some TFs but not their targets were shared between areas and some TFs-DEGs pairs were shared between areas, although sometimes the target could be up or downregulated for the different areas. Mostly, they were involved with cellular processes such as cell growth, cell survival, cell proliferation and/or differentiation, cell death, immune and inflammatory response and apoptosis **(**Table 3**)**. Different DMRs from CN and PT are localized in a binding site of the TF *REST*, with its target *CACNA1H* being down-regulated in both areas. *CACNA1H* was also targeted by *EGR1*, another TF that had a binding site in DMRs from both areas. In other cases, different DMRs in two brain areas, i.e. CN and OFC, were located in the binding site of the same TF, including *SP1*, that target *CD44* which was downregulated in OFC but upregulated in CN. There were also exclusive TFs for all brain areas **(**Additional file 2**: Table S7)**.

**Table 3:** Transcription factors (TFs) and targeted differentially expressed genes (DEGs) shared between the five brain areas

Brain areas	Targeted DEG	TF binding to DMR	Function ^†^
**ACC/ OFC**	*HLA-DPB1*	*RFX5* *(DNA-binding protein RFX5)*	Activates transcription from class II MHC promoters; Mediates cooperative binding between RFX and NF-Y.
**NAC/ CN**	*HLA-DQA1*		
	*HLA-DQB1*		
**CN/ PT**	*HLA-DRA*		
**OFC/ CN**	*CD44*	*SP1* *(Transcription factor Sp1)*	Activate/repress transcription in response to physiological and pathological stimuli; Binds with high affinity to GC-rich motifs and regulates the expression of a large number of genes involved in a variety of processes such as cell growth, apoptosis, differentiation and immune responses; Highly regulated by post-translational modifications; May have a role in modulating the cellular response to DNA damage; Implicated in chromatin remodeling.
**OFC/ CN/ PT**	*EGR1*		
**NAC/ CN**	*ABCC3*		
	*IGFBP3*		
**CN/ PT**	*HMGA1*		
	*KCNH2*		
**OFC/ PT**	*EGR1* *HES1*	*RELA* *(Transcription factor p65)*	Part of the NF-kappa-B; NF-kappa-B is a pleiotropic transcription factor present in almost all cell types and is the endpoint of a series of signal transduction events that are initiated by a vast array of stimuli related to many biological processes such as inflammation, immunity, differentiation, cell growth, tumorigenesis and apoptosis; NF-kappa-B homodimeric RELA-RELA complex appears to be involved in invasin-mediated activation of IL-8 expression.
	*HES1*	*RUNX3* *(Runt-related transcription factor 3)*	Bind to the core site of a number of enhancers and promoters, including murine leukemia virus, polyomavirus enhancer, T-cell receptor enhancers, LCK, IL3 and GM-CSF promoters; May be involved in the control of cellular proliferation and/or differentiation.
**NAC/ PT**	*HBB*	*CEBPB* *CCAAT/enhancer-binding protein beta)*	Regulate the expression of genes involved in immune and inflammatory responses; Its functional capacity is governed by protein interactions and post-translational protein modifications; Binds to regulatory regions of several acute-phase and cytokines genes and plays a role in the regulation of acute-phase reaction and inflammation.
		*NFYA* *(Nuclear transcription factor Y subunit alpha)*	Component of the sequence-specific heterotrimeric transcription factor (NF-Y) which specifically recognizes a 5′-CCAAT-3′ box motif found in the promoters of its target genes; NF-Y can function as both an activator and a repressor, depending on its interacting cofactors.
		*NFYB* *(Nuclear transcription factor Y subunit beta)*	Component of the sequence-specific heterotrimeric transcription factor (NF-Y) which specifically recognizes a 5′-CCAAT-3′ box motif found in the promoters of its target genes; NF-Y can function as both an activator and a repressor, depending on its interacting cofactors.
**CN/ PT**	*HMGA1*	*E2F1* *(Transcription factor E2F1*	Binds DNA cooperatively with DP proteins through the E2 recognition site; Function in the control of cell-cycle progression from G1 to S phase; It can mediate both cell proliferation and TP53/p53-dependent apoptosis.
	*CACNA1H*	*EGR1* *(Early growth response protein 1)*	Transcriptional regulator; Binds double-stranded target DNA, irrespective of the cytosine methylation status; Plays a role in regulating the response to growth factors, DNA damage, ischemia, regulation of cell survival, proliferation and cell death
	*CACNA1H*	*REST* *(RE1-silencing transcription factor)*	Transcriptional repressor which binds neuron-restrictive silencer element (NRSE) and represses neuronal gene transcription in non-neuronal cells; Maintains repression of neuronal genes in neural stem cells, and allows transcription and differentiation into neurons by dissociation from RE1/NRSE sites of target genes; Involved in maintaining the quiescent state of adult neural stem cells and preventing premature differentiation into mature neurons; Function in stress resistance in the brain during aging; possibly by regulating expression of genes involved in cell death and in the stress response.
	*GFAP*	*STAT3* *(Signal transducer and activator of transcription 3)*	Signal transducer and transcription activator that mediates cellular responses to interleukins, KITLG/SCF, LEP and other growth factors; Acts as a regulator of inflammatory response by regulating differentiation of naive CD4+ T-cells into T-helper Th17 or regulatory T-cells.
	*HLA-DRA*	*YY1* *(Transcriptional repressor protein YY1)*	Multifunctional transcription factor that exhibits positive and negative control on a large number of cellular and viral genes by binding to sites overlapping the transcription start site; Its activity is regulated by transcription factors and cytoplasmic proteins that have been shown to abrogate or completely inhibit YY1-mediated activation or repression.

^†^*Resumed from UniProt Knowledgebase (**https://www.uniprot.org/**)*

**Fig. 3:**
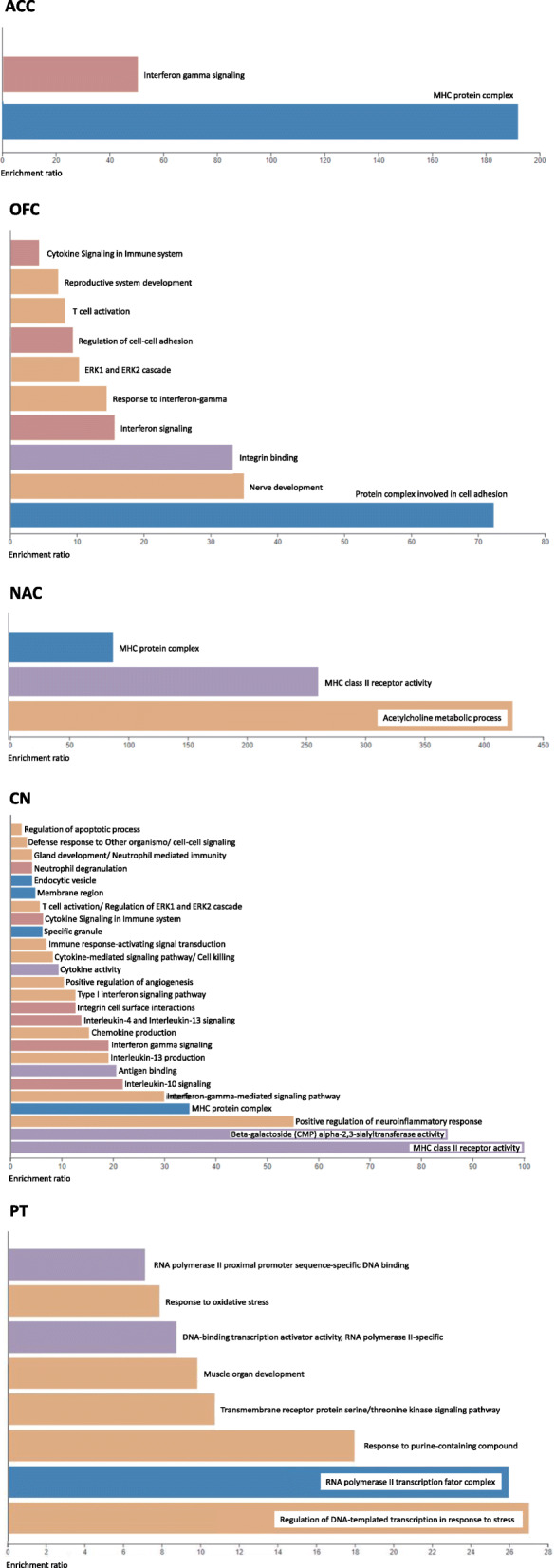
Enrichment results for Regulatory Networks for TFs from DMRs and targeted DEGs for the five brain areas

Targeted DEGs were submitted to WebGestalt [23] revealing enrichment for immune response processes for all areas **(**Fig. 3**)**. Regarding specific enrichments, we highlight interferon gamma signaling for ACC, ERK cascade for OFC, acetylcholine process for NAC, interleukin signaling for CN and regulation of DNA-templated transcription in response to stress for PT.

### DNA methylation age

**Fig. 4:**
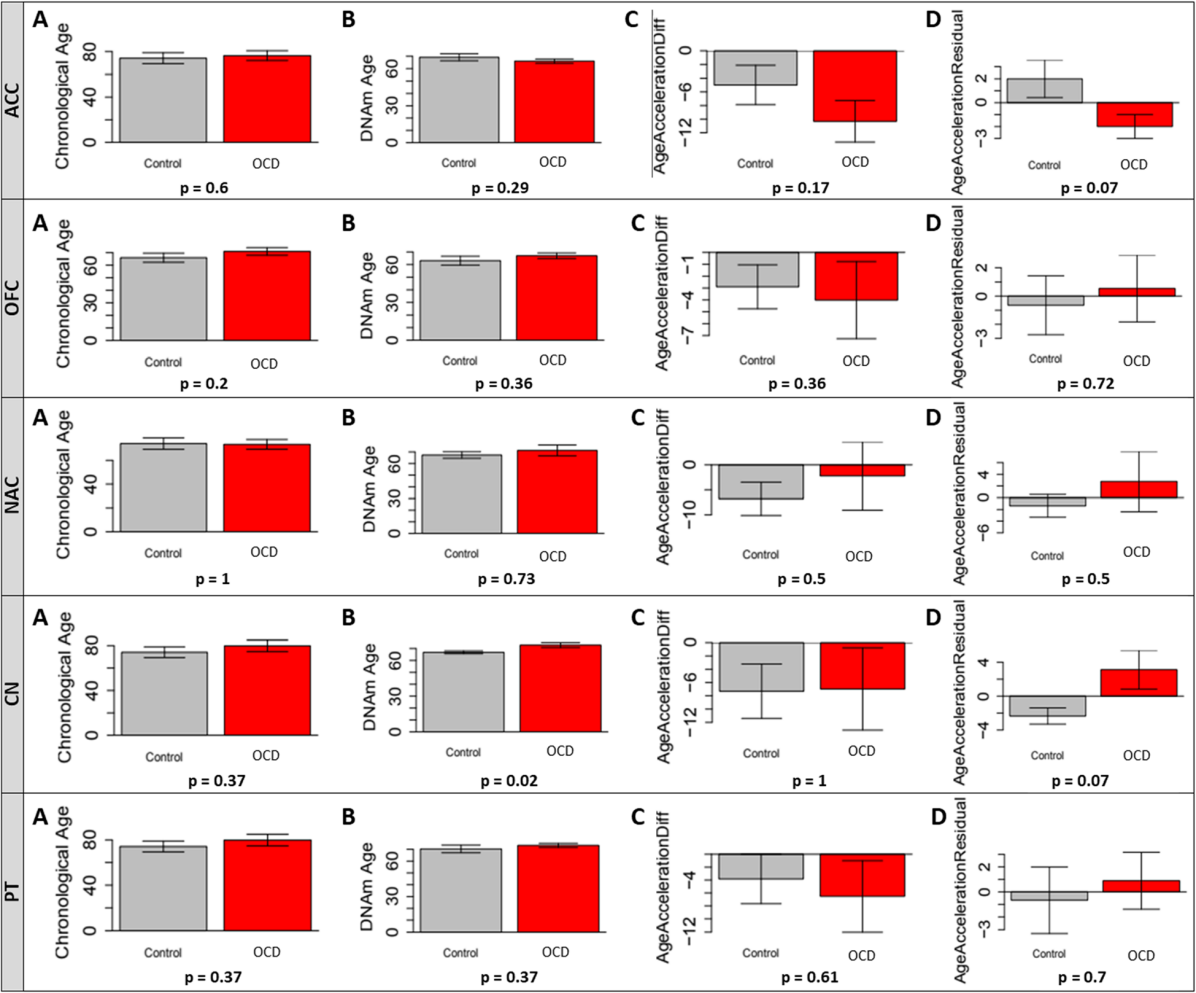
Comparison of chronological and DNAm age for each brain area from OCD and control groups. A: Chronological age; B: DNAm age; C: AA Difference; D: AA Residuals

We investigated the methylation age variations according to Horvath’s method [18]. In agreement with the chronological age, OFC, NAC, CN and PT from OCD had the DNAm age older than the respective areas of the control group. Only ACC presented DNAm age slightly younger than the chronological age for the OCD group. Although both AA difference and AA residuals presented a higher aging trend for the OCD group for almost all areas, the comparisons between OCD and control groups were not significant **(**Fig. 4**)**.

## Discussion

We explored DNA methylation and transcriptome data in post-mortem brain tissue associated with obsessive-compulsive disorder, searching for differences in the molecular mechanisms comparing OCD patients and controls. Regarding transcriptome analysis, there are some studies using blood and brain tissues, although for methylome analysis previous evidence comes from only peripheral assessment as blood [13, 14]. Due to the small sample size in our study, we searched DNA methylation differences using three approaches based on the assumptions that underlie DNA methylation studies: DMS, DMRs and gene modules as well as an integrative analysis of methylome and transcriptome data **(**Additional file 1**: Fig. S2)**.

We did not find DMSs with a corrected *p*-value for any brain area, not an unexpected result considering our sample size. CpG sites with p-value < 0.0005 and methylation differences ≥20% between OCD and controls, *ADARB2, UGT2B15, HLA-DQB1* and *HLA-DQA1* were recurrently identified by the different DNA methylation approaches suggesting that these genes have a role in OCD. A DNA methylation study in saliva of OCD patients reported that differentially CpG sites were only evident when more symptomatic cases were used [24].

Gene networks from methylation and gene expression integrated data pointed to the involvement of the G-protein signaling pathway and small GTPase signal transduction. Components from the GPCR pathway are expressed at different levels in all physiological systems, including the nervous and immune systems [25]. GPCRs signaling regulate, among others, the actin–cytoskeleton dynamic by activating small GTPases [26]. Several genes from the actin binding processes that are modulated by epigenetic regulation [27] were associated with OCD in peripheral blood of patients [13]. Actin binding maintains and modulates dendritic spines, growth cone and axon guidance [28, 29]. Axons and dendrites contain a specialized transcriptome capable of producing synaptic proteins independently of the cell soma [30]. In our study, we observed enriched processes for neuronal and glial structure, synapse compounds or signal transduction. Usually, synaptic inputs reach neurons via dendrites, in a postsynaptic position. This information is processed by cellular machinery and the output goes to the axon, arriving at the presynaptic area [31]. In all brain areas, we identified enriched processes for presynaptic and postsynaptic alteration, and related to parts of these both mechanisms suggesting again that the quality of synapses, gap junctions and consequently receptors and information transmission could be altered as a consequence of the disruption of DNAm in the individuals with OCD.

A member of the GPCRs family, the GABAB receptor, was identified in CN. GABAB receptors produce slow and prolonged inhibitory signals via G proteins, interact with several neurotransmitter receptors and regulate receptor activity. These receptors are broadly expressed in the nervous system and both GABAB pre and postsynaptic reduce neurotransmitter release and hyperpolarizing neurons, respectively. Altered GABAB receptor function has been related with neurological and psychiatric disorders [32, 33]. Also, GABAB has a functional crosstalk with N-methyl-D-aspartate (NMDA), an ionotropic glutamate receptor enriched for CN. NMDA has a role in activity-dependent changes in synaptic plasticity [32]. Glutamate receptors are involved with physiological and pathological processes in the central nervous system as well as the efficiency of synaptic transmission [34, 35] and are associated with OCD [36], with direct and indirect pathways operating on CSTC circuitry [37]. Moreover, individuals with OCD have higher GABA levels as well as GABA/glutamate ratio compared to health controls [38].

Enriched processes identified in NAC not found in the other brain areas were related to acetylcholine receptor activity and cholinergic synaptic transmission. An animal model showed that cholinergic interneurons’ activity in NAC were associated with adaptive cue-motivated behavior, present in many psychiatric conditions [39]. Acetylcholine seems to not interfere with signal attenuation in the cholinergic system of compulsive behavior in a rat model of OCD [40]. Nevertheless, in pediatric obsessive-compulsive disorder, specifically in children with PANDAS (Pediatric autoimmune neuropsychiatric disorders associated with streptococcal infections), cholinergic interneurons activity in the striatum may contribute to pathophysiology in children with rapid-onset OCD symptoms [41].

Additionally, we explored the potential involvement of transcription factors that corroborated the previous findings, such as the involvement of the immune system, replicated in all enrichment analysis for all brain areas. Immunological and neuroinflammatory alterations have been associated with psychiatric disorders, such as OCD. The role of the immune system in the pathophysiology of OCD is mediated by different processes, i.e. interleukin signaling, interferon gamma and MHC receptor activity [42, 43], also replicated in our analysis. Regarding the involvement of cytokines in OCD, they affect the central nervous system by altering neurotransmitter systems. We observed enriched pathways for the anti-inflammatory cytokines IL-17 for PT and IL-4, IL-5, IL-10, IL-13 for CN, reinforcing the role of cytokines in the pathogenesis of OCD [44]. The proinflammatory cytokine interferon gamma (IFN-γ) was also altered in CN and OFC. MHC receptor activity involves genes that control polymorphic proteins from the immune system [55], including HLA-DPB1 that was associated with OCD in a GWAS study comparing OCD cases, healthy controls and combined parents-child trios [45]. Here, HLA-DPB1 appears as down-regulated in ACC and OFC.

Some cognitive functions, such as learning and memory, and behaviors, such as feeding and locomotory, were enriched in our analysis for ACC, OFC, NAC and PT. Also, for OFC, synaptic assembling included *BDNF* among the genes, whose role was related to brain volume by neuroimaging as well as cognition outcomes, including social behavioral changes in OFC-amygdala circuit [46, 47]. There is an increasing amount of evidence supporting the association between *BDNF* and OCD phenotype and neurobiology [16, 48, 49]. Although it is still unclear how OCD symptoms/dimensions might be related to the immune alterations, some inflammatory processes were associated with the psychopathology of OCD by compromising cognitive functions.

We also identified an enrichment for the MAPK cascade, which is related to innate and adaptive immune response [50]. This pathway is involved in the regulation of conservative mechanisms in eukaryotic cells, including apoptosis and cell differentiation [51], and can coordinate and respond differently to stimuli including hormones and receptors [52]. ERK-MAPK pathway mediates several mechanisms involved in the pathogenesis of OCD. Specifically, the TrkB/ERK-MAPK pathway overactivation contributes to restoring normal behavior in an animal model of OCD. Moreover, up-regulation of the Ras/ERK-MAPK pathway was involved in the development of OCD-related disorders [53]. ERK and RAS cascade reactions signal to MAPKs, activating signal transduction, transcription factors and regulating gene expression. ERK cascade, which was enriched in our integration analysis for OFC, CN and PT, is activated by different stimuli, such as G protein-coupled receptors. RAS, the most prominent member of the small GTPase family, is activated after receptor phosphorylation, propagating signals that result in the activation of sequential kinases, including ERK [33, 54]. Along with RAS, other small GTPase presented enrichment in our results in CN, the Rho protein signal transduction was enriched in NAC. RHO regulates cytoskeleton reorganization, cell cycle progression and MAP kinase signal transduction. Proteins from the Rho GTPase family are expressed in the central nervous system and involved in cytoskeletal plasticity, especially in the actin cytoskeleton, modulating cell and axon migration [33]. Other pathways involved with Ras/MAPK, such as PI3K and cAMP signaling pathways. PI3K interacts with RAS as one of its main effector pathways and cooperates with MAPK pathways inducing DNA synthesis [54]. The regulation of PI3K activity through insulin-related signaling was described as a key player in OCD etiology, affecting dendritic spine and synapse formation [55]. cAMP signaling, also enriched in ACC, OFC, CN and PT, regulates the activation of MAPKs and also plays the opposite role, blocking the MAPK pathway through the binding of Raf-1 to Ras [54]. cAMP levels and activity was altered in patients with OCD compared to controls [56]. cAMP also plays a structural role in dendritic spines and in the structural enlargement of spines. Additionally, postsynaptic cAMP mechanism enhances structural potentiation of spines playing a key role in the regulation of structural synaptic plasticity [29].

Finally, the immune response has a close relation with stress response [57], biological processes that were enriched in all brain areas except for PT. Stress conditions play an important role in disrupting neurotransmission, synaptic plasticity, and cognitive functions such as memory and learning [57, 58]. An increasing amount of evidence of accelerated epigenetic age in some patients with neurodegenerative, psychiatric and cardiovascular diseases has been previously described [18, 19, 59]. In agreement to the chronological age, we observed older DNAm age for OCD group, as well as a higher aging trend regarding AA difference and AA residuals, which also agrees with the enrichment finding of apoptotic processes for all areas, aging enriched for OFC, CN and PT, and cellular senescence enriched for PT. Although not significant, it was noteworthy that in disagreement with other areas, ACC was the only region that showed negative age acceleration (AA) residual and presented a younger DNAm age for OCD group. A positron emission tomography study reported inflammation at OCD’s neurocircuitry in several brain areas, including OFC, CN, PT, thalamus and ventral striatum, but not ACC [60]. In ACC, reduced glutathione levels were found with aging and brain atrophy in mice with *EAAC1* mutation. *EAAC1* is a transporter of cysteine, which is a precursor for neuronal glutathione synthesis [61]. In addition, glutamatergic system gene variants were associated with lower concentrations of glutamate in the CSTC circuitry, particularly ACC, of patients with OCD [62, 63].

It is important to note that as a case-control study, our findings could be a result of the disease rather than a cause. As psychiatric disorders present high comorbid and genetic correlations [64], possibly our findings are not exclusive for OCD, although we identified genes previously associated with OCD and used strict inclusion and exclusion criteria for patients selection, seeking to ensure high quality and reduce biases as best as possible [65]. Also, considering limitations associated with small sample size and *post-mortem* collection of clinical information, our results must be viewed as a preliminary contribution regarding brain methylation patterns in OCD.

## Conclusions

Our findings confirm the involvement of previously associated genes and biological processes in OCD as well as report differences related to specific brain areas. These findings point to a role of cellular communication, inflammatory processes and behavior mediated by DNA methylation in OCD brain tissues. The main findings were related to the immune system, reaffirming the current literature findings about its involvement with OCD. We conclude that changes in DNA methylation are involved with OCD and further studies are needed to characterize alterations in different paths in each brain area.

## Methods

### Participants and OCD clinical assessment

Brain samples were retrieved from the Sao Paulo Autopsy Service from the University of São Paulo as part of the psychiatric disorders collection of the Biobank for Aging Studies. Three psychiatrists performed a screening interview encompassing clinical, functional, cognitive and psychiatric parameters (detailed in [66]) to a family member or close caregiver who had at least weekly contact with the deceased. For individuals with a presumably diagnosis of OCD, a second clinical evaluation with the same informant was done for the confirmation of the diagnosis. The complete evaluation comprised the Structured Clinical Interview for DSM IV Axis I disorder (SCID) [67], the Yale- Brown Obsessive-compulsive scale (Y-BOCS) [68] and a short version of DY-BOCS [69]. The questionnaire was modified to ask the questions about the deceased case. As standard procedure in the second evaluation, informants showed the medication (or medication boxes) that were used. After the second assessment, a best estimated diagnosis procedure was done by two psychiatrists [9, 65]. Briefly, from 109 individuals, 72 were assigned to the psychiatric group, being 22 diagnosed with OCD by clinical parameters and 50 diagnosed with other psychiatric disorders (i.e. bipolar disorder, major depression, Tourette syndrome, schizophrenia and others). Most individuals were unmedicated to psychiatric medications. Thirty-seven individuals did not fulfill psychiatric diagnosis criteria. Of the 22 OCD cases, eight had the best estimated diagnosis and were included in this study **(**Additional file 1**: Table S8)**. Eight controls without any psychiatry diagnosis, matched by age, sex and brain’s hemisphere were selected from the same Biobank. All individuals were 50 years of age or older, without a history of clinical dementia and no other clinical comorbidity which could result in hypoxia or brain autolysis (as high postmortem hour, hospitalization with mechanical breathing, chronic obstructive pulmonary disease, kidney failure and previous cerebrovascular accident). These individuals were considered as healthy controls in the study. Brain tissues from both OCD cases and healthy controls were collected from cortico-striatal areas ACC, OFC, NAC, CN and PT **(**Additional file 1**: Fig. S3).** More detailed information concerning sample collection and the complete evaluation can be found elsewhere [9, 65, 70].

### DNA methylation data

#### DNAm profiling and data quality control

Brain samples were dissected to isolate the cortico-striatal areas and preserved in cryotubes at − 80 °C. DNA was extracted using QIAsymphony DNA Kit (Qiagen, Hilden, Mettmann, Germany) on QIAsymphony platform, according to the manufacturer’s instructions. DNA was bisulfite-converted using the EZ DNA Methylation kit (Zymo Research, Irvine, California, USA), according to manufacturer’s instructions and hybridized in the Illumina Infinium HumanMethylation450 BeadChip array (Illumina Inc., San Diego, California, USA). Raw data were extracted by the iScan SQ scanner (Illumina) using GenomeStudio software (v.2011.1), with the methylation module v.1.9.0 (Illumina), into IDAT files, which were imported to R statistical environment using minfi package [71]. Quality control steps removed 16,217 probes associated with SNPs (Single nucleotide polymorphism) (that contained SNPs at the CpG interrogation site or at the single nucleotide extension), 10,007 probes with unreliable measurements (*p* > 0.05), 26,357 probes located in specific contexts (non-CpG sites) and 10,871 probes located in the sexual chromosomes. All samples passed quality control parameters resulting in 422,060 probes to be analyzed. Background was corrected using the noob method [72] and cell composition was estimated using *FlowSorted.DLPFC.450 k* function [73] as implemented in the minfi package [71]. Cell composition was evaluated for each brain region considering the groups to be compared. We used the *compareGroups* package [74] that pointed to OFC as presenting differences in cell composition. Thus, cell composition was considered as a variable to be corrected only when OCD cases and health controls were compared for the OFC region. ChAMP package was used to identify and correct batch effects and biological variables (sex) [75, 76]. Distribution of Infinium I and II probes fluorescence measurements was normalized by functional normalization (FunNorm) method [77].

#### Identification of DNAm changes related to OCD and methylation clock

For each brain region, we applied the linear model function from limma [78] to M-values (loggit of B-values) to identify differentially methylated CpG sites (DMSs) [79]. As parameters, we considered adjusted *p*-value (adjP) ≤0.05 after multiple testing corrections using the Benjamini and Hochberg method. Age was included as a variable for all brain areas and cellular composition was included only for OFC. As multiple CpG sites may map to the same gene, and the effect of DNAm can be dependent on its location in relation to the gene (promoter, body), we used Functional Epigenetic Modules (FEM) analysis. The FEM package [80], as implemented in ChAMP, identifies subnetworks (protein interaction modules - PPI) where a significant number of members exhibit differential DNAm in relation to the phenotype of interest. To identify differentially methylated regions (DMRs), DMRcate was applied to the methylation values [81], considered significant those with FDR (False Discovery Rate) < 0.05 and methylation differences (delta beta, Δβ) ≥10%. DNAm age was calculated with the beta values using Horvath’s method [18]. The algorithm also calculated de age acceleration (AA), which can be used to determine how fast tissues are aging, i.e., whether the DNAm age of a given tissue is consistently higher (or lower) than expected [18, 82].

### Transcriptome data

The list of differentially expressed genes (DEG) comparing OCD cases and controls from NAC, CN and PT was retrieved from a previously published study by our group [9]. For ACC and OFC, RNASeq data were preprocessed using the same parameters [9]. ACC dataset was composed of 8 controls and 8 OCD samples and OFC for 5 and 6 controls and OCD samples, respectively. Any ribosomal RNA that bypassed the depletion process from the read counts of both the ACC and OFC regions was removed. To filter genes that were lowly expressed, we converted the read counts to counts per million (CPM) values using the edgeR library (v.3.28.1) [83], choosing only transcripts that had at least 0.3 CPM in 50% for a group (controls or OCD). For both datasets, we used the DESeq2 library (v.1.26) [84] to normalize the raw counts with the *rlog* function and the surrogate variable analysis (SVA) library (v.3.34) [85] to estimate any hidden covariates, using sex, age, laboratory batch and OCD diagnostic as our variables of interest. The DESeq2 were also used to perform the differential expression analysis, using as covariates in the linear model the same variables of interest with the addition of surrogate variables estimated by the SVA for each brain region (3 surrogate variables for the ACC and 2 surrogate variables for the OFC).

### Bioinformatic integrative analysis

#### Integrative analysis considering DEGs and methylation differences (CpG sites mapped to genes)

For the five brain areas, we merged the three datasets (DMR and FEM genes list from methylation analysis and DEGs from the RNASeq analysis) and use the STRING Database [86] to construct a PPI network for each brain area, where, we used all active interaction sources with our list of genes, selecting only the edges with a high confidence score (≥0.7). From this PPI network, we selected edges that connected nodes from 2 different sources (DMR, DEG or FEM lists), to remove nodes that were exclusively connected with its own DMR, DEG or FEM dataset and to guarantee that for each PPI network, we had the major connected component integrating both transcriptomic and methylation data. To calculate network centralities metrics, we used the NetworkX library (v.1.9.1), choosing the metrics: Degree - evaluate the number of connections from each node; Neighbor degree - the number of the degrees from each node’s immediate neighbor; Closeness - also a measure of the influence of a node in the network, but based on its node’s positioning; Eigenvector - measure the global influence from a node [87]; KATZ centrality - a variation from the Eigenvector centrality used on social networks, capable to measure both the local and the global influence of a node; Betweenness - measures the number of paths between a pair nodes that cross a given node. A gene high betweenness might be capable to connect isolated elements from a network; Clustering Coefficient - measure the number of triangles in the network, in other words, the number of immediate neighbors that are also connected between each other [88]. The selected nodes were re-submitted to STRING [86] (using the same parameters) and PPI enrichment analysis was applied to evaluate if the brain region networks have more interactions, considering both GO and REACTOME, non-redundant ontologies/pathways with a minimum of 5 genes.

#### Integrative analysis considering combination between transcription factors annotated from DMRs (not mapped to genes) and DEGs

After identifying the DMRs, they were annotated for transcription factors (TFs) using ENCODE (Encyclopedia of DNA Elements) [22], *wgEncodeRegTfbsClusteredV3* track available from Genome Browser’s Data Integrator [89]. To match the TFs associated with the DMRs to the DEGs previously identified for our dataset [9], we used the TRRUST v2 database [90]. Additionally, annotated TFs and its targets were used to construct regulatory networks, indicating the type of connection (activation, repression or unknown interaction). Targeted DEGs were submitted to WebGestalt [23] for GO and REACTOME enrichment analysis, considering only non-redundant ontologies with a minimum of 5 genes related and FDR < 0.05.

## Supplementary Information

**Additional file 1: Fig. S1.** Functional modules of subnetworks of connected genes identified with FEM analysis. **A:** Cortical areas – Modules 1–5 from anterior cingulate gyrus (ACC) and modules 1–9 from orbitofrontal cortex (OFC); B: Striatal areas – Modules 1–5 from nucleus accumbens (NAC), modules 1–3 from putamen (PT) and modules 1–8 from caudate nucleus (CN). DNA methylation level is represented by color intensity. **Fig. S2.** Summary of designed experiments**.** A: Evaluation of DNA methylation (DNAm) and transcriptome in post-mortem brain tissues of the anterior cingulate gyrus (ACC), orbitofrontal cortex (OFC), nucleus accumbens (NAC), caudate nucleus (CN) and putamen (PT) from OCD patients and matched controls; B: DNAm characterization resulting in DNA methylation age (DNAm), Functional Epigenetic Modules (FEM) and differentially methylated regions (DMRs). Differentially expressed genes (DEGs) generated by transcriptome analysis: NAC, CN and PT were retrieved from a previously published study by our group [10] and ACC/OFC were processed for the present study; C: Methylation and gene expression integration data using genes from DMRs, FEM modules and DEGs with supplemental enrichment analysis considering both Gene ontology (GO) and REACTOME pathways; D: Integration analysis considering combination between transcription factors annotated from DMRs (not mapped to genes) and DEGs with supplemental enrichment analysis considering both GO and REACTOME pathways. **Table S8.** Description of obsessive–compulsive symptoms of the OCD patients. **Fig. S3.** Relation between individuals and related brain areas.

**Additional file 2: Table S1.** Differentially methylated sites (DMSs) between OCD cases and controls. **Table S2.** Functional modules identified by FEM (Functional Epigenetic Modules) analysis. **Table S3.** Differentially methylated regions (DMRs) and related cgs. **Table S4.** Differential expression analysis between OCD cases and controls. **Table S5.** Nodes (genes) from STRING final networks classified according to network centrality measures. **Table S6:** Enrichment results from STRING for the final networks using differentially expressed genes (DEG), genes located on differentially methylated regions. **Table S7.** Transcription factors (TF) annotated from differentially methylated regions (DMRs) matched on differentially expressed genes (DEGs).

## Data Availability

The datasets generated during the current study are available in the GEO repository under Accession Number GSE148021 (https://www.ncbi.nlm.nih.gov/geo/query/acc.cgi?acc=GSE14802) and SRA repository under accession number and SRP127180 (https://www.ncbi.nlm.nih.gov/bioproject/?term=PRJNA421175). All data generated in this study are included in the Supplementary Information.

## Abbreviations

AA: Age acceleration
ACC: Anterior cingulate gyrus
adjP: Adjusted *p*-value
CN: Caudate nucleus
CpG: Cytosine-phosphate-guanine
CSTC: Cortico-striato-thalamo-cortical circuitry
DEG: Differentially expressed genes
dlPFC: Dorsolateral prefrontal cortex
DMRs: Differentially methylated regions
DMSs: Differentially methylated CpG sites
DNAm age: DNA methylation age
DNAm: DNA methylation
ENCODE: Encyclopedia of DNA Elements
FDR: False Discovery Rate
FEM: Functional Epigenetic Modules
GO: Gene Ontology
NAC: Nucleus accumbens
OCD: Obsessive-compulsive disorder
OFC: Orbitofrontal cortex
PPI: Protein interaction modules
PT: Putamen
SNPs: Single nucleotide polymorphism
SVA: Surrogate variable analysis
TFs: Transcription factors
TSS: Transcription start sites
Δβ: Delta beta
