# Supplementary material for: Brain areas involved with obsessive-compulsive disorder present different DNA methylation modulation
Source: BMC Genom Data. 2021 Oct 30;22:45. doi: 10.1186/s12863-021-00993-0 (PMC8557022; doi:10.1186/s12863-021-00993-0)
Supplement: Supplementary file 1 — Additional file 1: Fig. S1. Functional modules of subnetworks of connected genes identified with FEM analysis. A: Cortical areas – Modules 1–5 from anterior cingulate gyrus (ACC) and modules 1–9 from orbitofrontal cortex (OFC); B: Striatal areas – Modules 1–5 from nucleus accumbens (NAC), modules 1–3 from putamen (PT) and modules 1–8 from caudate nucleus (CN). DNA methylation level is represented by color intensity. Fig. S2. Summary of designed experiments. A: Evaluation of DNA methylation (DNAm) and transcriptome in post-mortem brain tissues of the anterior cingulate gyrus (ACC), orbitofrontal cortex (OFC), nucleus accumbens (NAC), caudate nucleus (CN) and putamen (PT) from OCD patients and matched controls; B: DNAm characterization resulting in DNA methylation age (DNAm), Functional Epigenetic Modules (FEM) and differentially methylated regions (DMRs). Differentially expressed genes (DEGs) generated by transcriptome analysis: NAC, CN and PT were retrieved from a previously published study by our group [10] and ACC/OFC were processed for the present study; C: Methylation and gene expression integration data using genes from DMRs, FEM modules and DEGs with supplemental enrichment analysis considering both Gene ontology (GO) and REACTOME pathways; D: Integration analysis considering combination between transcription factors annotated from DMRs (not mapped to genes) and DEGs with supplemental enrichment analysis considering both GO and REACTOME pathways. Table S8. Description of obsessive–compulsive symptoms of the OCD patients. Fig. S3. Relation between individuals and related brain areas. [file 12863_2021_993_MOESM1_ESM.docx]

Brain areas involved with obsessive-compulsive disorder present different DNA methylation modulation

Kátia Cristina de Oliveira ^1,2,3^, Caroline Camilo ^1^, Vinícius Daguano Gastaldi ^1^, Arthur Sant’Anna Feltrin ^2^, Bianca Cristina Garcia Lisboa ^1^, Vanessa de Jesus Rodrigues de Paula ^1^, Ariane Cristine Moretto ^3^, Beny Lafer ^1^, Marcelo Queiroz Hoexter ^1,4^, Euripedes Constantino Miguel ^1,4^, Mariana Maschietto ^5^, Biobank for Aging Studies Group* & Helena Brentani ^1,4^

^1^ Departamento & Instituto de Psiquiatria, Faculdade de Medicina FMUSP, Universidade de Sao Paulo, Sao Paulo, Brazil. ^2^ Center of Mathematics, Computation and Cognition, Federal University of ABC, São Bernardo do Campo, Brazil. ^3^ Faculdade de Medicina FMUSP, Universidade de Sao Paulo, Sao Paulo, Brazil. ^4^ Laboratório de Psicopatologia e Terapêutica Psiquiátrica (LIM23), Faculdade de Medicina FMUSP, Universidade de Sao Paulo, Sao Paulo, Brazil. ^5^ Centro de Pesquisa, Centro Infantil Boldrini, Campinas, Brazil. These authors contributed equally: Kátia Cristina de Oliveira, Caroline Camilo. *A list of authors and their affiliations appears at the end of the paper.

**Additional file 1**

**S1 Figure A
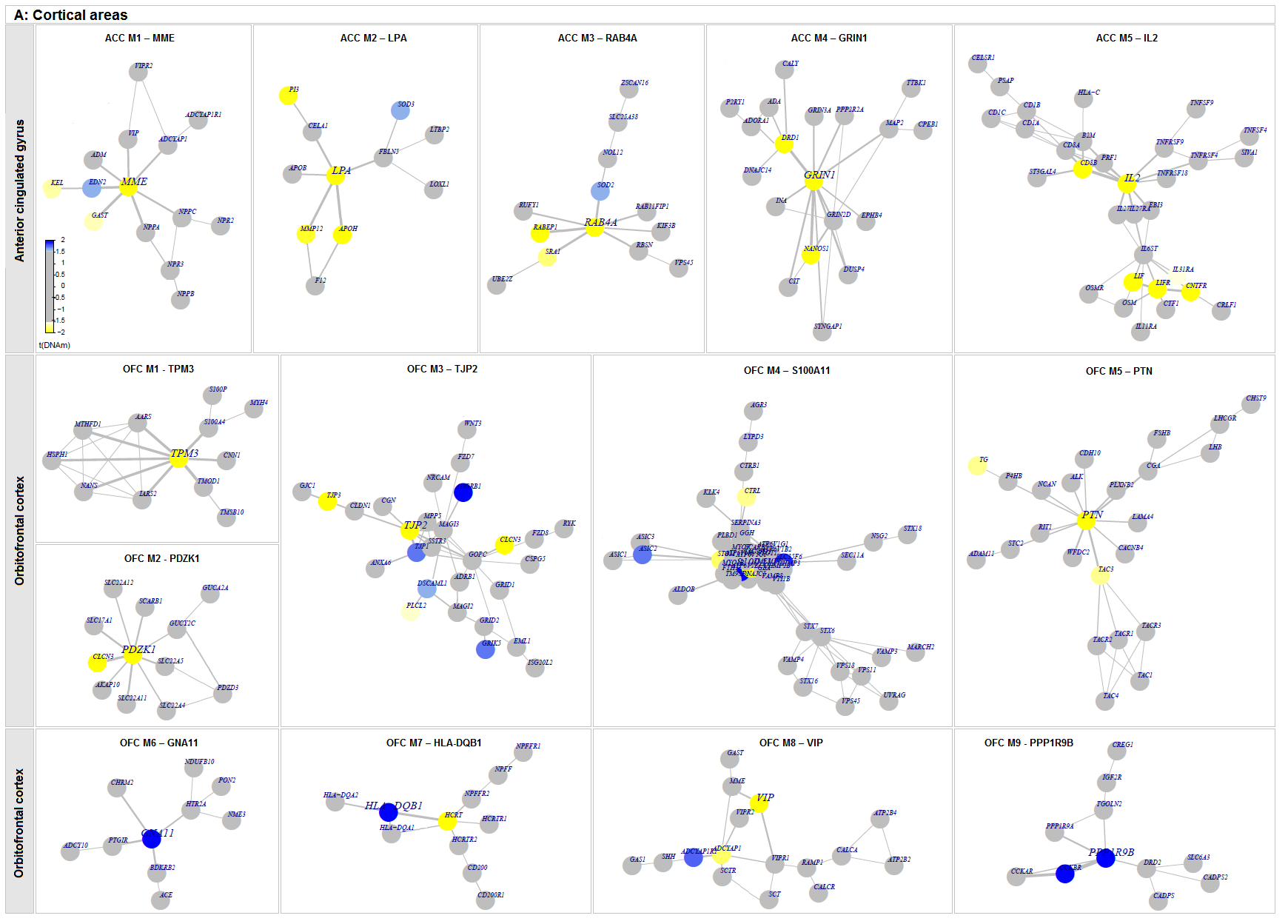
**

**S1 Figure B
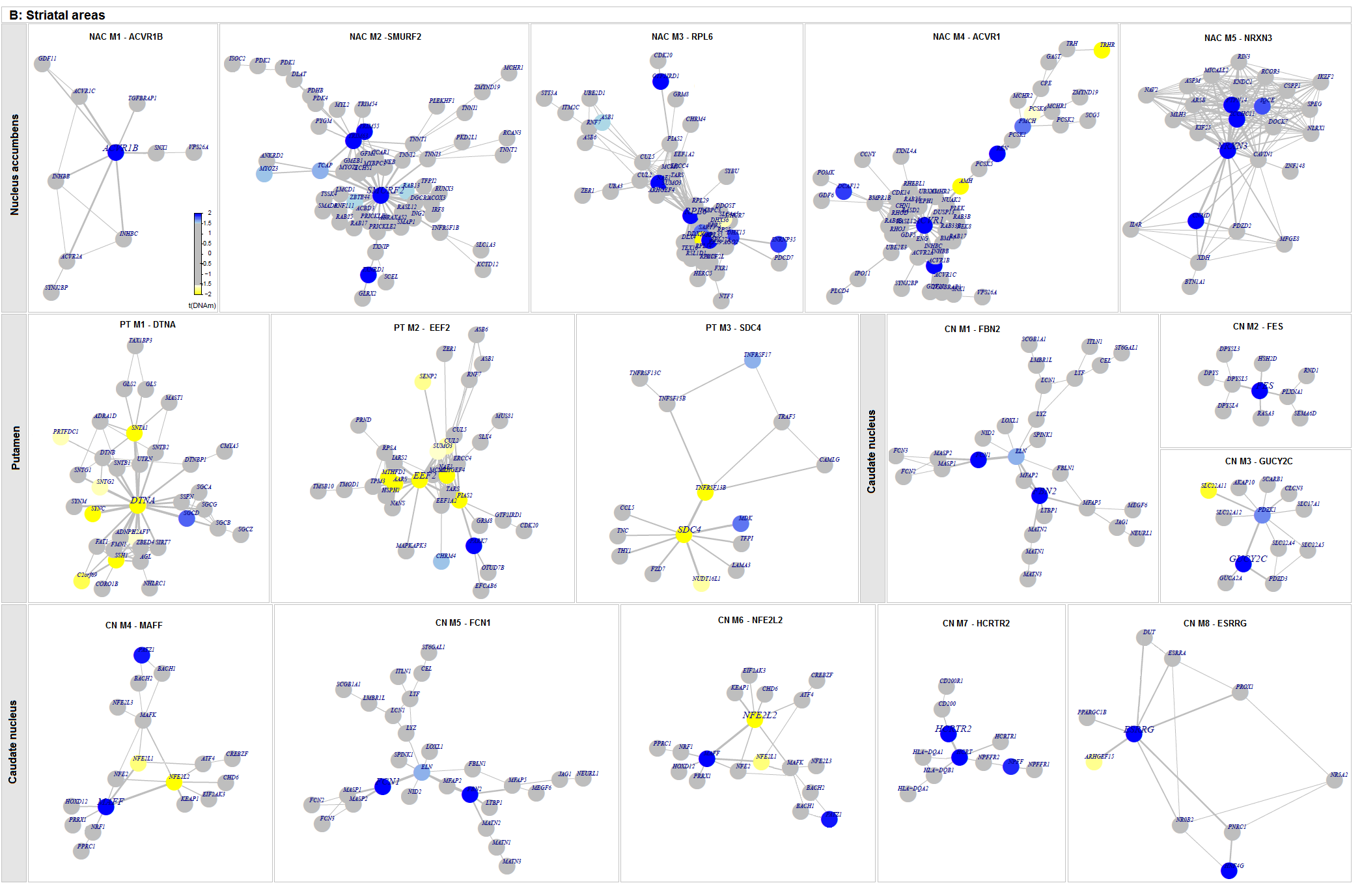
 S1 Figure:** Functional modules of subnetworks of connected genes identified with FEM analysis. **A:** Cortical areas – Modules 1-5 from anterior cingulate gyrus (ACC) and modules 1-9 from orbitofrontal cortex (OFC); B: Striatal areas – Modules 1-5 from nucleus accumbens (NAC), modules 1-3 from putamen (PT) and modules 1-8 from caudate nucleus (CN). DNA methylation level is represented by color intensity.

**
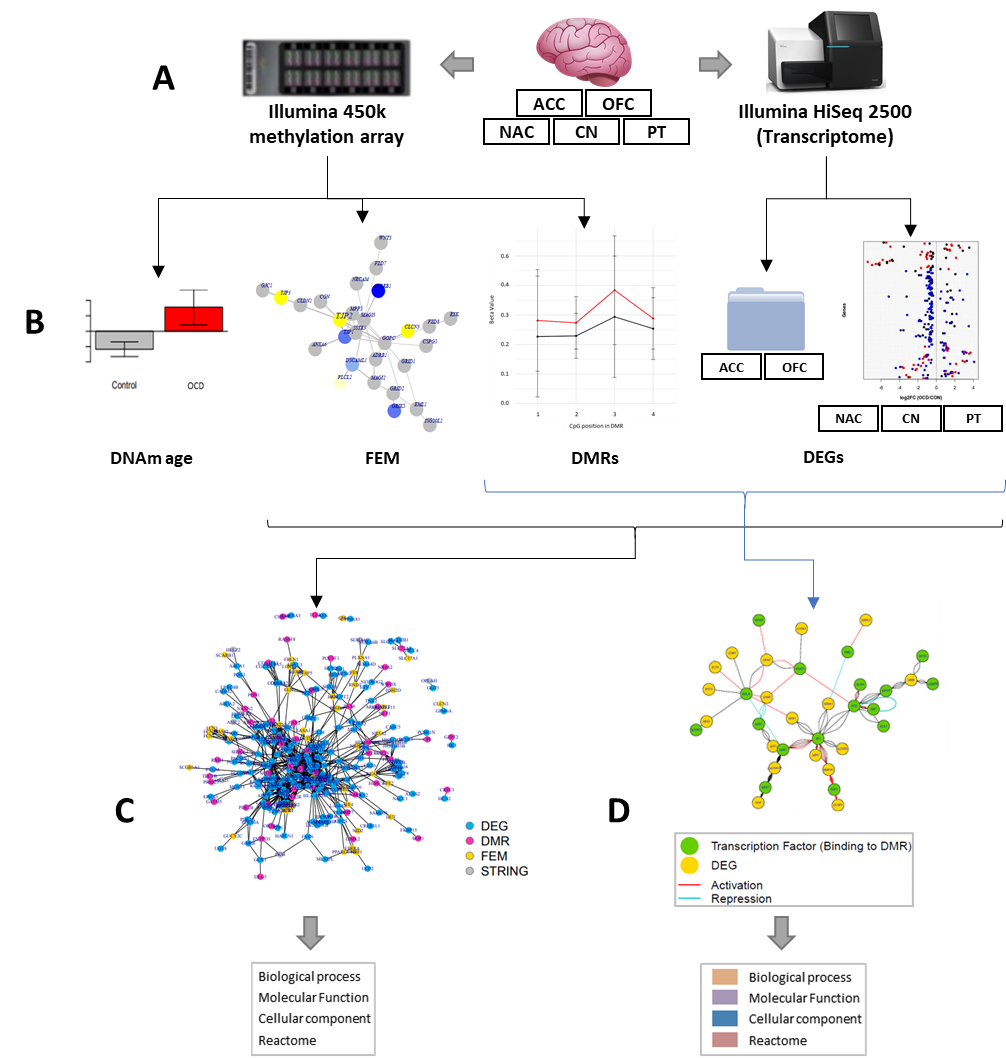
**

**S2 Figure:** Summary of designed experiments**.** A: Evaluation of DNA methylation (DNAm) and transcriptome in post-mortem brain tissues of the anterior cingulate gyrus (ACC), orbitofrontal cortex (OFC), nucleus accumbens (NAC), caudate nucleus (CN) and putamen (PT) from OCD patients and matched controls; B: DNAm characterization resulting in DNA methylation age (DNAm), Functional Epigenetic Modules (FEM) and differentially methylated regions (DMRs). Differentially expressed genes (DEGs) generated by transcriptome analysis: NAC, CN and PT were retrieved from a previously published study by our group (10) and ACC/OFC were processed for the present study; C: Methylation and gene expression integration data using genes from DMRs, FEM modules and DEGs with supplemental enrichment analysis considering both Gene ontology (GO) and REACTOME pathways; D: Integration analysis considering combination between transcription factors annotated from DMRs (not mapped to genes) and DEGs with supplemental enrichment analysis considering both GO and REACTOME pathways.

**S8 Table:** Description of obsessive–compulsive symptoms of the OCD patients.

| **ID** | **Sex** | **Age** | **Sort of OCD symptoms** | **NPI behavioral disturbances** | **CDR** | **IQCODE** | **SCIDD** | **SCIDM** |
| --- | --- | --- | --- | --- | --- | --- | --- | --- |
| OCD-01 | Male | 66 | Collecting/hoarding; contamination/cleaning; ‘just-right’ perceptions | none | 0 | 3 | Yes^3^ | No |
| OCD-02 | Male | 77 | Contamination/cleaning; compulsions to count and motor tics | Hallucinations^1^; aberrant motor behavior; night-time behavior disturbances | 0 | 3 | No | No |
| OCD-03 | Female | 66 | Obsessions about symmetry; ‘just-right’ perceptions; hoarding | Night-time behavior disturbances; appetite and eating abnormalities | 0 | 3 | Yes^4^ | No |
| OCD-04 | Male | 81 | Ordering/arranging; aggression/disaster; compulsions of hoarding | none | 0 | 3 | No | No |
| OCD-05 | Female | 99 | Compulsions of checking; contamination/cleaning; ordering/arranging; hoarding | Delusions^2^; agitation; aberrant motor behavior; night-time behavior disturbances; appetite and eating abnormalities | 0 | 3 | No | No |
| OCD-06 | Male | 86 | Contamination/cleaning; obsessions about disasters; compulsions of checking | Night-time behavior disturbances; appetite and eating abnormalities | 0 | 3 | No | No |
| OCD-07 | Male | 74 | Obsessions about symmetry; ‘just-right’ perceptions; ordering/arranging; hoarding | Agitation, dysphoria, anxiety, apathy, night-time behavior disturbances, and appetite and eating abnormalities | 0 | 3 | No | No |
| OCD-08 | Female | 62 | Obsessions of disasters; obsessions about symmetry; ‘just-right’ perceptions; ordering/arranging; miscellaneous obsessions/compulsions that relate to superstitions; hoarding | none | 0 | 3 | No | No |

NPI: Neuropsychiatry Inventory - ^1^: Information very unspecific. Family reported that the subject used to talk to himself (mild frequency and severity). Subject never had any diagnosis of psychotic disorder in life; ^2^: Information very unspecific relatives. Family reported that the subject used to say that people wanted to steal him. Subject has never had any diagnosis of psychotic disorder in life; CDR: Clinical Dementia Rating; IQCODE: Informant Questionnaire on Cognitive Decline in the Elderly; SCIDD: Structured clinic interview for depression - ^3^: Family reported that the subject did not use psychiatric medication; ^4^: Family was unable to inform about psychiatric medication use; SCIDM: Structured clinical interview for mania.

*Adaptad from de Lisboa et al., 2019.*


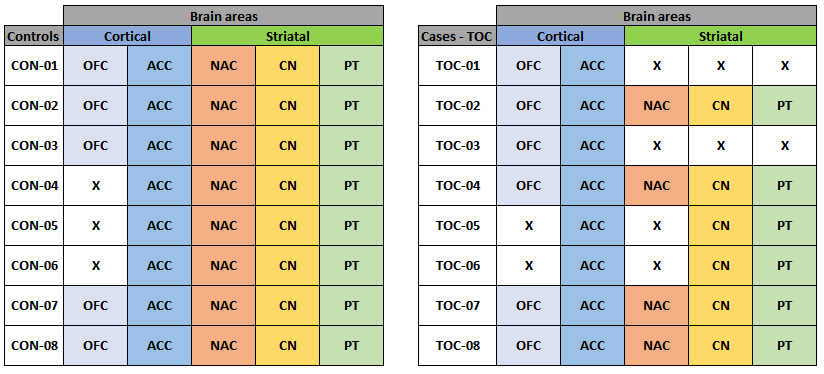


**S3 Figure:** Relation between individuals and related brain areas.
